# Ultra-Processed Food Consumption and Depressive Symptoms in a Mediterranean Cohort

**DOI:** 10.3390/nu15030504

**Published:** 2023-01-18

**Authors:** Justyna Godos, Marialaura Bonaccio, Wahidah H. Al-Qahtani, Wolfgang Marx, Melissa M. Lane, Gian Marco Leggio, Giuseppe Grosso

**Affiliations:** 1 Department of Biomedical and Biotechnological Sciences, University of Catania, 95123 Catania, Italy; 2 Department of Epidemiology and Prevention, IRCCS NEUROMED, 86077 Pozzilli, Italy; 3 Department of Food Sciences & Nutrition, College of Food & Agriculture Sciences, King Saud University, Riyadh 11451, Saudi Arabia; 4 Food & Mood Centre, The Institute for Mental and Physical Health and Clinical Translation (IMPACT), School of Medicine, Deakin University, Geelong, VIC 3220, Australia; 5 Center for Human Nutrition and Mediterranean Foods (NUTREA), University of Catania, 95123 Catania, Italy

**Keywords:** ultra-processed foods, NOVA classification, food processing, nutritional psychiatry, depression, depressive symptoms

## Abstract

Excess consumption of ultra-processed foods (UPFs) is currently under investigation for its potentially detrimental impact on human health. Current evidence demonstrates a substantial association with an increased risk of metabolic disorders, but data on mental health outcomes are just emerging. The aim of this study was to investigate the relationship between the consumption of UPFs and depressive symptoms in a sample of younger Italian adults. A cross-sectional study was conducted on 596 individuals (age 18–35 y) recruited in southern Italy. Food frequency questionnaires and the NOVA classification were used to assess dietary factors; the Center for the Epidemiological Studies of Depression Short Form (CES-D-10) was used to assess presence of depressive symptoms. Individuals in the highest quartile of UPF consumption had higher odds of having depressive symptoms in the energy-adjusted model (odds ratio (OR) = 1.89, 95% confidence interval (CI): 1.06, 3.28); the association remained significant after adjusting for potential confounding factors (OR = 2.04, 95% CI: 1.04, 4.01) and became even stronger after further adjustment for adherence to the Mediterranean diet as a proxy of diet quality (OR = 2.70, 95% CI: 1.32, 5.51). In conclusion, a positive association between UPF consumption and likelihood of having depressive symptoms was found in younger Italian individuals. Given the consistency of the findings after adjustment for diet quality, further studies are needed to understand whether non-nutritional factors may play a role in human neurobiology.

## 1. Introduction

Dietary risk factors have been accounted to be responsible for about 10 million deaths due to cardiovascular diseases, metabolic disorders, and certain cancers in 2017 [1]. The issues related to overnutrition, especially in developed countries, depend on several factors that vary from personal choices to exposure to an obesogenic environment, from societal decisions to industry inputs [2]. Younger generations are at the highest risk, being registered as those under the strongest environmental pressure driven by stressful modern lifestyle, lack of time leading to scarce physical activity, poor sleep, and unhealthy behaviors, including low quality dietary habits [3]. In this regard, the globalization of food markets, the growing inputs from the food industry (in terms of quality of available food products), and a general hardship in financial situation are known to promote “Westernized-type diets”, as opposed to traditional dietary patterns characterized by minimally-processed, locally produced, plant-based foods [4]. All together, these factors lead to various levels of malnutrition, including low-quality dietary patterns characterized by highly-palatable, convenient, energy-rich, and nutrient-poor foods [5].

Studies investigating the level of processing as a potential variable of interest to predict health outcomes are nowadays using the so-called NOVA classification to identify a category of foods classified as “ultra-processed”. Based on the NOVA classification, ultra-processed foods (UPFs) are foods characterized by formulations containing few or no natural ingredients, supplemented with chemical additives and preservatives to prolong shelf life, but also supply intense palatable features and properties (i.e., flavor enhancers, colorants, emulsifiers, artificial sweeteners, thickeners, and foaming/anti-foaming agents) [6]. UPFs are widely consumed in modern societies, although with large differences across countries [7]. Studies show that UPF intake may range from 15–20% of daily energy intake in Mediterranean countries, to up to 80% in US, UK, Canadian, and Australian populations, with a substantially higher rate of consumption among younger individuals [8]. This variation may significantly affect their impact on the general population, as well as the projection of the future burden of disease related to the consumption of UPFs.

There is an ongoing debate about whether the negative health effects of UPFs stem from poor nutritional quality of food processing [9]. Contrary to the idea that UPFs might exert a negative impact on health due to the poor nutritional content, we recently demonstrated that higher risk of mortality associated with high UPF intake was independent from the nutritional quality of the diet [10]. The concerns regarding the consumption of UPFs rely on the observed association with various non-communicable diseases, such as obesity, cardiometabolic diseases, and lately also behavioral disorders [11,12]. Among the most under-studied research topics, diet has been hypothesized to be an independent risk factor for mental disorders [3]. There is growing evidence that various dietary factors may be associated with depressive symptoms, although the nature and direction of this relation are largely unknown [13]. Several hypotheses and mechanisms have been proposed [14,15], which suggest that inflammatory processes related to food intake may explain part of the relationship between dietary factors and brain health [16]. Among the various aspects of the diet that may exert such suggested effects, UPFs are currently under investigation for a potential negative impact toward mental health outcomes [17,18]. The aim of this study was to investigate whether an association between UPF consumption and depressive symptoms could be observed in a cohort of southern Italian younger adults.

## 2. Materials and Methods

### 2.1. Study Design and Population

The present study is a cross-sectional analysis of the baseline data from the Mediterranean healthy Eating, Aging and Lifestyle (MEAL) study, an observational study aiming to explore the relation between lifestyle behaviors and non-communicable diseases in a population recruited in the Mediterranean area [19]. Participants were randomly selected between 2014 and 2015 in the main districts of Catania, in southern Italy. The recruitment and data collection were performed through the registered records of local general practitioners stratified by sex and 10-year age groups. Out of 2405 individuals invited, the final sample included 2044 participants with a response rate of 85%. For the purposes of this study, data from individuals under 35 years old were included (*n* = 735). The goals of the project have been described to the participants prior to acceptance of participation by written informed consent. All the study procedures were conducted in accordance with the Declaration of Helsinki (1989) of the World Medical Association. The study protocol has been reviewed and approved by the concerning ethical committee.

### 2.2. Background Data

Face-to-face, computer assisted interviews were conducted by trained personnel to collect data on sex, age, educational (the highest educational degree achieved) and occupational (the most important employment during the year before the investigation or before retirement) statuses, smoking status, and physical activity level. Marital status was categorized as (i) unmarried/widowed or (ii) married. Educational status was categorized as (i) low (primary/secondary), (ii) medium (high school), and (iii) high (university). Occupational status was categorized as (i) unemployed, (ii) low (unskilled workers), (iii) medium (partially skilled workers), and (iv) high (skilled workers). The International Physical Activity Questionnaires (IPAQ) [20] were used to evaluate physical activity level and categorized as (i) low, (ii) moderate, and (iii) high. Smoking status was categorized as (i) non-smoker, (ii) ex-smoker, and (iii) current smoker. Eating habits included questions on skipping breakfast, snacking habits, and skipping dinner, with answers categorized as (i) always/often and (ii) seldom/never.

### 2.3. Dietary Information and UPF Calculation

Validated instruments were used to collect data on dietary consumption over the previous year [21,22]. The food frequency questionnaire (FFQ) included questions on average consumption of 110 foods and beverages with nine response options ranging from “never” to “4–5 times per day”. For food items generally consumed over certain periods of the year, the questions referred to seasonal consumption and results were proportionally adjusted. The instrument demonstrated an acceptable relative validity and reliability when validated for the Italian population [21]. Nutrient (macro- and micro-) and non-nutrient (polyphenol) dietary content was estimated by calculating the 24 h intake of foods and beverages (in gr or ml, respectively) and estimating the correspondent daily intake of nutrients from the food composition tables of Council for Research in Agriculture and Analysis of Agricultural Economy (CREA) [23]. Data entries with lacking information or unreliable intakes (<1000 or >6000 kcal/d) were excluded from the analyses (*n* = 52) leaving a total of 683 individuals included in the analysis.

To provide indication of overall diet quality, adherence to the Mediterranean diet was used as a proxy. The literature-based score [24,25] takes into account the daily consumption of food groups that are considered as key features of the Mediterranean diet, providing positive points for increasing portions (up to 2 points) of fruit, vegetables, legumes, cereals, fish, and olive oil, negative points for increasing portions of meat and dairy foods, and positive points for moderate alcohol intake. The score is ultimately composed of the summary points from 0 to 18 points, with higher scores indicating higher adherence to the Mediterranean diet. For the purposes of this study, the sample was grouped in tertiles and categorized as (i) low, (ii) medium, and (iii) high adherence to the Mediterranean diet.

UPF consumption was calculated by applying the NOVA classification to the major food groups consumed in the study sample [26]. Briefly, 110 food items of the long FFQ were classified as follows: group 1, unprocessed or minimally processed foods (i.e., rice and other cereals, meat, fish, milk, eggs, fruit, vegetables, nuts, etc.); group 2, processed culinary ingredients (i.e., sugar, vegetable oils and butter); group 3, processed foods (i.e., processed breads and cheese); group 4, UPFs (i.e., confectioneries, salty snacks, fast-foods, soft drinks, etc.) [27]. For the purpose of this study, the mean share of the NOVA group 4 (UPFs) to the total daily energy intake was estimated, and participants were categorized into quartiles of energy shares of UPFs as the variable of exposure.

### 2.4. Depressive Symptoms

Screening for depressive symptoms was performed using the 10-item Center for the Epidemiological Studies of Depression Short Form (CES-D-10) [28]. Briefly, the CES-D-10 is a self-administered tool that includes 10 questions commonly used to test for presence of depressive symptoms in the general population. The frequency of mood/symptoms during the previous week is rated by a 4-point Likert scale ranging from 0 indicating rarely or none of the time (less than 1 day) to 3 indicating most or all of the time (5–7 days). The total score is calculated by summing up the scores of the individual questions and ranges from 0 to 30, with higher scores indicating greater severity of symptoms; conventionally, a score >15 indicates the presence of depressive symptoms. After excluding individuals with incomplete or unreliable questionnaires (*n* = 87), a total sample of 596 was included in the final analysis.

### 2.5. Statistical Analysis

Categorical variables are presented as frequencies of occurrence and percentages, with a Chi-squared test used to assess differences between quartiles of UPF consumption. Continuous variables are expressed as mean and standard deviations (SDs), with ANOVA test used to test differences between groups. The association between UPF consumption and presence of depressive symptoms was tested by logistic regression analyses through calculation of odds ratios (ORs) and 95% confidence intervals (Cis) for an energy-adjusted model, a multivariate model adjusted for baseline characteristics (age, sex, educational and occupational status, smoking, and physical activity level, marital status, and snacking habits), and a third model with additional adjustment for adherence to the Mediterranean diet (as a proxy for diet quality). All reported *p* values were based on two-sided tests and compared to a significance level of 5%. SPSS 21 (SPSS Inc., Chicago, IL, USA) software was used for all the statistical calculations.

## 3. Results

The distribution of baseline characteristics by quartiles of UPF consumption in the study sample is presented in Table 1. There was a significantly different distribution of UPF consumption by marital status (*p* = 0.006) and physical activity level (*p* = 0.004), although with no clear trend across categories, but a tendency of higher consumption in unmarried and medium/highly physically active individuals. In contrast, individuals consuming more UPFs reported significantly lower adherence to the Mediterranean diet, with opposite trends in those reporting lower consumption (*p* < 0.001).

When testing for differences in major food groups, micro- and macro-nutrients across quartiles of UPF consumption, most of the macronutrients, sodium, total and processed means were consumed significantly more by those in the highest quartile of UPF intake. In contrast, fiber and certain food groups such as cereals, fruits, vegetables, legumes, dairy products, and olive oil were consumed less among individuals in the highest quartile of UPF intake (Table 2).

Table 3 shows the association between UPF consumption and presence of depressive symptoms. Individuals in the highest quartile of UPF consumption had higher odds of having depressive symptoms in the energy-adjusted model (OR = 1.89, 95% CI: 1.06, 3.28); the association remained significant after adjusting for potential confounding factors (including age, sex, energy intake, educational and occupational lever, smoking status, eating habits, and physical activity level) (OR = 2.04, 95% CI: 1.04, 4.01) and became even stronger after further adjustment for adherence to the Mediterranean diet (OR = 2.70, 95% CI: 1.32, 5.51) (Table 3).

## 4. Discussion

This study provides cross-sectional evidence of an association between higher UPF consumption and an increase in depressive symptoms. Furthermore, in contrast to the hypothesis that UPF may affect mental health due to the poor nutritional quality of the diet, further adjustment for adherence to the Mediterranean diet (as a proxy of diet quality) increased, rather than reduced, the association between UPF consumption and presence of depressive symptoms, suggesting that components of the diet other than nutritional quality may play a role on the reported association.

Two recent meta-analyses, including mostly cross-sectional investigations, reported that higher consumption of UPFs is associated with increased depressive symptoms [17,18]. With specific reference to the association between UPF consumption and depression, one of the first studies published on this topic has been conducted on about 26,000 French participants within the NutriNet-Santé cohort reporting an average 32% daily energy intake from UPFs; the authors found that a 10% increase in %UPF in the diet was associated with a 21% higher risk of depressive symptoms over a 5-year follow-up period [29]. Another study involving nearly 15,000 Spanish university graduates (mean age 36.7 years) participating in the “Seguimiento Universidad de Navarra” (SUN) Project, reported a 33% higher risk of depression in high UPF consumers (about 400 g/d) after a follow-up of 10 years [30]. In addition, studies with higher mean intake of UPFs reported similar findings. The National Health and Nutrition Examination Survey (NHANES) including nearly 14,000 US adults (with an average 55% of total energy intake from UPFs) showed that individuals in the highest quartile of UPF consumption were more likely (43% higher odds) to have depressive symptoms, compared to the lowest category of consumption [31]. An updated report from the same sample revealed that individuals with the highest level of UPF consumption were significantly more likely to report at least mild depression, more mentally unhealthy and more anxious days per month [32]. Although not specifically quantifying the intake of UPFs, a study conducted on about 3500 participants showed that individuals consuming a “processed food” dietary pattern characterized by high intake of sweetened desserts, fried food, processed meat, refined grains, and high-fat dairy products were more likely to have depressive symptoms compared to those with less consumption [33]. Similar associations have been reported for broader mental health conditions in other cohorts. In a sample of nearly 3000 Brazilian adolescents, higher consumption of UPFs has been associated with higher rates of internalizing symptoms including depression and anxiety [34]. In addition, data from the Adolescent School-Based Health Survey including nearly 100,000 adolescents showed that daily UPF consumption and sedentary behaviors were associated with higher odds for anxiety-induced sleep disturbance [35], which was mediated by loneliness and eating while watching TV or studying [36]. Finally, a cross-sectional study conducted on 1270 Brazilian retail workers showed that UPF consumption was associated with high perceived stress levels [37]. Most studies reported some sort of pattern of background characteristics associated with high UPF consumption: younger age, being unmarried/living alone, frequent out-of-home eating, often high cultural level. In line with our findings, this data suggest that younger individuals might be a more susceptible group of the population at higher risk of mood disorders due to a number of potential factors (work-related stress, lack of time, financial instability, etc.). This suggests that the rise in UPF consumption may be driven not only due to their highly palatable nature, but also due to economic and practical convenience.

From a mechanistic point of view, several hypotheses have been suggested and supported by scientific literature to explain the detrimental effects of UPF consumption on mental health outcomes. UPF consumption, as well as various dietary factors, may affect systemic inflammation with a consequently higher risk of non-communicable diseases, including mental disorders [38,39]. High UPF consumption has been demonstrated to be characterized by a rise in intake of refined sugars (such as high-fructose corn syrup) and saturated/trans fatty acids, accompanied with lower intake of fiber [7]. The high energy density of UPFs may lead to an imbalance of regulation and homeostatic maintenance of cells, causing an impairment of their microenvironment and finally compromising their functionality and integrity [40]. High intracellular glucose derived from high-free sugar food products increases intermediate metabolites of oxidative metabolism, mitochondrial dysfunction, and subsequently increases reactive oxygen species (ROS) production [41]. Similarly (albeit with totally different mechanisms), a high consumption of saturated and trans fatty acids induces a suffering of the endoplasmic reticulum at an intracellular level, modification of cellular membranes, and activation of transcription factors related to oxidative stress and proinflammatory pathways, including nuclear factor kB, related to the production of proinflammatory cytokines and the mTOR, JNK, and AKT pathways [42]. Finally, high consumption of UPFs has been reported to be often associated with lower intake of fiber, which may represent an additional mechanism related to disruption of homeostasis, immune regulation, and establishment of mental health issues [43]. Specifically, high UPF consumption as well as a lack of dietary fiber may induce an imbalance of the gut microbiota and lead to dysbiosis [44]; this condition is characterized by changes in their functional composition and metabolic activities, including a reduction in short chain fatty acids (SCFAs) and a rise in lipopolysaccharides producing bacteria, intestinal barrier dysfunction, and bacterial translocation into the bloodstream, tissues, and organs causing systemic immune system activation and inflammation [45]. Moreover, gut microbiota may communicate with the central nervous system through interaction with enteroendocrine and enterochromaffins cells, which are able to transmit signals via vagal or afferent nerve fibers and induce responses into the brain (i.e., serotonin release) [46]. Besides this indirect mechanism of central nervous system involvement, gut microbiota modifications also impact gut peptides and hormones (i.e., neuropeptide Y, glucagone-like peptide-1, cholecystokinin, ghrelin, corticotropin-releasing factor) which are all involved, to a various extent and through different mechanisms, in the complex gut–brain axis communication [47]. Long-term exposure to highly-palatable UPFs, and production of pro-inflammatory cytokines and secondary products of oxidative stress at brain level may also play a role in the alteration of the physiological feeding patterns, leading to food-anticipatory and binge-type behaviors, potential failure in self-control [48,49], which in turn are associated with anxiety/depressive symptoms [50] and alteration of sleep quality [51].

Concerns also arise regarding food additives that have been shown to exert neurotoxicity and clinical manifestation of depression, cognitive decline, and eating disorders [52]. Common food additives are generally used in UPFs for a variety of purposes, including in the alteration of organoleptic properties such as non-caloric sweeteners, flavor and color enhancers, emulsifiers, foaming/anti-foaming and anti-caking agents [53]; these compounds have been shown to affect human physiology in various ways, including oral processing, alteration of the gut microbiota homeostasis, uncoupling between predicted calories and consequent response from the digestive system, and further development of oxidative stress and the pro-inflammatory actions as the main mechanisms of toxicity [54]. The promotion of inflammatory processes associated with the consumption of UPFs may potentially affect the functioning of common neuronal signaling systems (i.e., serotonergic and dopaminergic systems) and of certain brain regions (i.e., amygdala) implicated in mental health disorders [55]. Some additives may contain nanoparticles that exert higher toxicity when compared to the bulk material because they are absorbed, cross various biological barriers, and may accumulate in tissues and organs [56]. These compounds may have direct interactions at the cellular level, exerting local toxicity by increasing the reactive oxygen and nitrogen species production, inducing mitochondrial and DNA oxidation, and activating pro-inflammatory cellular pathways [57]. Finally, the transformation processes of UPFs may lead to the production of substances, such as acrylamide, acrolein, polycyclic aromatic hydrocarbons, and furan that are known to be toxic to the human organism [58]. These compounds have been shown to potentially exert neurotoxicity via microbiota–gut–brain axis signaling and inflammasome-related neuroinflammation [59,60,61].

The findings of this study should be considered in light of some methodological limitations. First, due to the observational nature of the study design, the results may be affected by reverse causation, and the study design does not allow us to define cause–effect relationships, only association. However, the findings from this study do not necessarily imply that UPFs must necessarily cause depression, but that a mutual relation may exist, that UPFs might be consumed as comfort foods by an at-risk population (i.e., younger individuals with emerging mood disorders), and that it can establish a vicious cycle by further enhancing detrimental effects on brain health related to depression. Second, although the most common potential confounding factors have been taken into account when adjusting for multivariate analyses, the existence of residual unmeasured confounders cannot be ruled out. Third, the assessment of dietary intakes (FFQ) is limited in its nature by recall bias, portion size uncertainty, and social desirability. Finally, the general low consumption of UPF, especially among older individuals, did not allow us to provide data for all age groups, and results for older participants were null possible due to lack of statistical power (data not shown); although it is important to distinguish the findings by age groups due to generational differences in exposure and risk factors, we missed the opportunity to generalize the results also among older groups of individuals.

## 5. Conclusions

In conclusion, a positive association between UPF consumption and likelihood of having depressive symptoms was found in younger southern Italian adults. Further studies are needed to corroborate this association, also among other populations. It is crucial to understand whether non-nutritional factors may also play a role in human neurobiology. The specific involvement of brain regions involved in behavioral disorders needs to be further investigated to better understand the impact of food additives on human mental health.

## Figures and Tables

**Table 1:** Baseline characteristics of the study sample according to quartiles of intake of UPFs (*n* = 596).

	UPF Consumption				*p*-Value
	Q1	Q2	Q3	Q4	
**Age, mean (SD)**	28.6 (5.7)	29.8 (6.0)	29.6 (5.5)	28.7 (5.9)	0.189
**Sex, *n* (%)**					0.699
Men	33 (40.2)	46 (35.7)	76 (42.0)	84 (41.2)	
Women	49 (59.8)	83 (64.3)	105 (58.0)	120 (58.8)	
**Marital status, *n* (%)**					0.006
Unmarried/widowed	51 (62.2)	68 (52.7)	114 (63.0)	146 (71.6)	
Married	31 (37.8)	61 (47.3)	67 (37.0)	58 (28.4)	
**Smoking status, *n* (%)**					0.587
Never	49 (59.8)	90 (69.8)	120 (66.3)	137 (67.2)	
Current	29 (35.4)	31 (24.0)	53 (29.3)	53 (26.0)	
Former	4 (4.9)	8 (6.2)	8 (4.4)	14 (6.9)	
**Educational level, *n* (%)**					0.059
Low	17 (20.7)	19 (14.7)	13 (7.2)	32 (15.7)	
Medium	35 (42.7)	49 (38.0)	79 (43.6)	82 (40.2)	
High	30 (36.6)	61 (47.3)	89 (49.2)	90 (44.1)	
**Occupational level, *n* (%)**					0.153
Unemployed	21 (28.8)	32 (27.8)	37 (25.7)	66 (39.8)	
Low	11 (15.1)	13 (11.3)	15 (10.4)	16 (9.6)	
Medium	16 (21.9)	19 (16.5)	35 (24.3)	25 (15.1)	
High	25 (34.2)	51 (44.3)	57 (39.6)	59 (35.5)	
**Physical activity level, *n* (%)**					0.004
Low	19 (23.2)	21 (16.3)	19 (10.5)	27 (13.2)	
Medium	28 (34.1)	72 (55.8)	85 (47.0)	110 (53.9)	
High	35 (42.7)	36 (27.9)	77 (42.5)	67 (32.8)	
**Eating habits, *n* (%)**					
Skipping breakfast	17 (20.7)	31 (24.0)	52 (28.7)	53 (26.0)	0.546
Daily snacking	12 (14.6)	17 (13.2)	23 (12.7)	26 (12.7)	0.975
Skipping dinner	9 (11.0)	12 (9.3)	22 (12.2)	34 (16.7)	0.220
**Adherence to Mediterranean diet, *n* (%)**					<0.001
Low	30 (36.3)	70 (54.3)	103 (56.9)	147 (72.1)	
Medium	46 (56.1)	46 (35.7)	44 (24.3)	50 (24.5)	
High	6 (7.3)	13 (10.1)	34 (18.8)	7 (3.4)	

**Table 2:** Mean (and standard deviation) consumption of micro-, macronutrients and major food groups intake according to quartiles of UFP consumption.

	UPF Consumption				*p*-Value
	Q1	Q2	Q3	Q4	
***mean (SD)***					
Energy intake (kcal/d)	2045.88 (873.58)	2067.57 (738.74)	2008.67 (821.52)	2283.99 (1105.62) *	0.019
Energy intake (kJ/d)	8230.25 (3651.25)	8378.72 (3084.12)	8186.26 (3446.88)	9275.7 (4593.52) *	0.023
**Macronutrients**					
Carbohydrates (g/d)	304.47 (141.20)	315.29 (124.31)	294.34 (128.04)	313.02 (152.21)	0.493
Fiber (g/d)	39.42 (31.57)	33.75 (15.72)	31.34 (15.98)	31.05 (19.5) *	0.008
Protein (g/d)	92.86 (53.88)	89.59 (34.09)	86.08 (40.07)	94.05 (47.70)	0.324
Fat (g/d)	54.51 (24.17)	55.69 (20.12)	59.07 (23.87)	77.84 (41.13) **	<0.001
Cholesterol (mg/d)	173.69 (129.47)	178.7 (80.11)	190.19 (112.56)	244.64 (131.42) **	<0.001
SFA	19.93 (8.74)	21.35 (8.02)	22.49 (8.60)	31.50 (16.31) **	<0.001
MUFA	23.74 (9.41)	24.07 (8.32)	25.23 (10.66)	31.15 (15.85) **	<0.001
PUFA	11.17 (5.89)	10.68 (4.41)	11.36 (7.11)	12.91 (8.23) *	0.019
Total Omega-3	1.73 (0.96)	1.66 (0,78)	1.57 (0.73)	1.61 (0.77)	0.509
**Micronutrients**					
Vitamin A (Retinol)	950.29 (571.75)	942.28 (449.58)	868.43 (425.27)	898.99 (491.21)	0.452
Vitamin C (mg/d)	195.19 (185.64)	178.35 (108.56)	164.96 (101.98)	155.92 (106.05)	0.062
Vitamin E (mg/d)	9.30 (4.67)	8.82 (3.53)	8.95 (3.85)	9.87 (5.74)	0.141
Vitamin B12	8.34 (18.02)	6.78 (4.56)	7.01 (6.56)	7.99 (6.79)	0.421
Vitamin D	5.29 (5.52)	5.60 (4.98)	5.94 (7.67)	6.19 (8.41)	0.769
Sodium (mg/d)	2890.45 (1136.79)	3151.52 (1158.34)	2953.90 (985.94)	3302.72 (1599.08) *	0.020
Potassium (mg/d)	4299.95 (3013.97)	3892.82 (1545.45)	3731.17 (1894.24)	3948.06 (2143.45)	0.243
**Foods**					
Cereals (g/d)	236.26 (144.97)	256.83 (139.76)	208.58 (112.36)	167.70 (121.70) **	<0.001
Vegetables (g/d)	334.55 (304.85)	283.70 (143.48)	257.64 (143,.60)	256.29 (170.06) *	0.006
Fruit (g/d)	506.21 (458.81)	409.42 (343.75)	406.27 (364.51)	371.04 (320.63) *	0.042
Legumes (g/d)	334.55 (304.85)	283.70 (143.48)	257.64 (143.60)	256.29 (170.06) *	0.009
Nuts (total, g/d)	17.05 (20.58)	16.64 (20.74)	18.35 (22.60)	25.44 (72.74)	0.253
Fish (g/d)	77.90 (120.38)	66.18 (66.88)	78.99 (116.29)	67.80 (88.37)	0.569
Eggs (g/d)	1.89 (3.49)	2.53 (5.12)	2.80 (5.40)	2.13 (4.33)	0.396
Meat (total, g/d)	76.71 (47.87)	74.43 (47.43)	66.97 (37.27)	77.03 (34.53) *	0.077
Red meat (g/d)	34.23 (22.52)	35.28 (25.67)	35.03 (31.88)	35.81 (22.68)	0.973
Processed Meat (g/d)	13.05 (12.45)	17.71 (15.67)	18.03 (14.09)	31.45 (31.46) **	<0.001
Dairy products (g/d)	216.86 (187,86)	238.17 (166,41)	176.12 (155,34)	155,33 (204.18) *	0.019
Alcohol (total, g/d)	8.15 (13.42)	5.06 (7.25)	5.43 (7.54)	6.30 (9.35)	0.078
Olive oil (ml/d)	7.10 (3.10)	7.15 (3.04)	6.44 (2.91)	6.22 (3.08) *	0.018

* indicates *p* <0.05 for ANOVA analysis, ** indicates *p* <0.001 for ANOVA analysis.

**Table 3:** Association between intake of UPFs and having depressive symptoms in the study sample.

	UPF Consumption			
	Q1	Q2	Q3	Q4
	OR (95% CI)			
Model 1	1	1.05 (0.56, 1.96)	1.17 (0.65, 2.09)	1.87 (1.06, 3.29)
Model 2	1	1.26 (0.62, 2.57)	0.93 (0.46, 1.89)	2.04 (1.04, 4.01)
Model 3	1	1.44 (0.70, 2.97)	1.17 (0.56, 2.43)	2.70 (1.33, 5.51)

Model 1 was adjusted for energy intake. Model 2 was further adjusted for age (mean), sex, marital status, educational level, occupational level, physical activity level, smoking status, eating habits. Model 3 was further adjusted for level of adherence to the Mediterranean diet.

## Data Availability

The data that support the findings of this study are available upon reasonable request.
